# An Intrinsically Transparent Polyamide Film with Superior Toughness and Great Optical Performance

**DOI:** 10.3390/polym16050599

**Published:** 2024-02-22

**Authors:** Jianlin Li, Yong Yi, Chunhua Wang, Weijian Lu, Mingxi Liao, Xin Jing, Wenzhi Wang

**Affiliations:** 1 National & Local Joint Engineering Research Center for Advanced Packaging Material and Technology, Hunan University of Technology, Zhuzhou 412007, China; lijianlin@stu.hut.edu.cn (J.L.); xx08200710@163.com (M.L.); 2 Key Laboratory of Advanced Packaging Materials and Technology of Hunan Province, Hunan University of Technology, Zhuzhou 412007, China

**Keywords:** transparent polyamide, crystalline behavior, optical performance, biaxially oriented films

## Abstract

Polyamide 66 was extensively utilized in various applications contributed by its excellent mechanical performance and outstanding durability. However, its high crystallinity renders it to have low transparency, which seriously limits its application in optical devices. Herein, a highly transparent polyamide (PA) 66-based copolymer was reported using 4,4′-diaminodicyclohexylmethane (PACM), adipic acid, and polyamide 66 salt as the reaction monomers. Wide-angle X-ray diffraction (WAXD) analysis revealed that the crystal phase of the synthesized PA66/PACM6 displayed a clear transition from *α* to *γ* as the PACM6 increased accompanied by a decreased intensity in the diffraction peak of the copolymer, whose transmittance was successfully adjusted reaching as high as 92.5% (at 550 nm) when the PACM6 was 40 wt%. Moreover, the copolymer with a higher content of PACM6 exhibited larger toughness. On the other hand, the biaxially oriented films of PA66/PACM6 (20 wt%) were also prepared, and it was found that the transparency of the PA66/PACM6 copolymer could be further enhanced via adjusting the stretching ratio of the film. Furthermore, the mechanical strength of the biaxially oriented PA66/PACM6 was also improved with the increase in the orientation degree in the stretching process, indicating that the physical properties of the transparent PA66 were significantly influenced by its alicyclic structure, and the introduction of PACM into PA66 was capable of effectively improving the optical and crystalline characteristics of PA66, revealing that the synthetic strategy has great potential for guiding the design and development of transparent polyamide materials.

## 1. Introduction

Polyamide (PA) 66, first reported in 1935, is a synthetic polymer that has gained widespread usage in various industries including automotive, electrical and electronics, machinery, and medical fields owing to its excellent performance, such as superior chemical resistance, high thermal stability, strong mechanical strength, and ease of processing [1]. The continued development and versatility of PA66 have firmly established it as a cornerstone in the engineering of plastics [2,3]. Transparent polyamide, a specialized form of polyamide engineered to provide optical transparency while retaining the advantageous properties of conventional polyamide [4,5], such as high strength, heat resistance, and chemical durability [6,7,8], has become increasingly sought after in design and engineering. Compared with other transparent materials, transparent polyamide not only combines the high transparency but also inherits the traditional polyamide’s unique advantages such as strong strength, long durability, and excellent chemical resistance. Therefore, it has been widely used in various industries, including automotive, consumer goods, medical, electronics, and aerospace [9,10,11].

Blending with transparent polymers is a common way to prepare the transparent polyamide-based materials, such as polycarbonate (PC), bacterial cellulose (BC), and polymethylmethacrylate (PMMA) [12,13,14,15,16,17]. During the processing, the degradation and yellowing were another problem for PAs, which were always mixed with UV stabilizers to enhance the transparency [18,19]. However, there was always a trade-off between the transparency and the mechanical performances of the polyamide via blending. The inherent performance of the polyamide matrix such as strength, wear resistance, or chemical resistance might be sacrificed after blending.

Besides blending, copolymerization is another approach to prepare transparent polyamide PA, which typically involves the polymerization of PA with other copolymer monomers with superior optical properties, and the resultant copolymer is always transparent [20,21]. For example, the bio-based 1,5pentylenediamine-2,5-furan dicarboxylic acid (FDCA) (PA 5F) and 1,10-decylenediamine (DA10)-FDCA(PA 10F) have been successfully prepared, which displayed not only high transparency but also good mechanical properties. These polyamides have been employed in producing pipes and household plastic equipment [10].

On the other hand, a series of transparent poly(hexamethylene isophthalamide/poly(m-benzoyl4,4′-methylenebis(cyclohexylamine)) (PA6I/PACMI) have also been successfully synthesized, which exhibited an essentially amorphous structure and high optical transparency with transmittance reaching up to 90% [22]. Moreover, a novel transparent engineering polyamide, which was PA 10I/10T (synthesized from poly(decamethylene isophthalamide) (PA10I), isophthalic acid (IPA), and DA10), has also been obtained with the transmittance ranging between 85.0% and 90.0% [23]. The key to copolymerization lies in selecting suitable copolymer monomers and adjusting copolymerization reaction conditions to achieve the desired transparency and other properties, such as strength, wear resistance, and chemical resistance. This method allows for more precise control over reaction conditions and formulation to ensure that the resulting material meets the expected performance standards.

Moreover, biaxial stretching is a popular technique to be utilized to produce packaging films, such as biaxially oriented polypropylene (BOPP), biaxially oriented polylactic acid (BOPLA), biaxially oriented polyethylene terephthalate (BOPET), and biaxially oriented polyamide (BOPA) [24,25,26,27]. It involves stretching the film in two directions, typically in the machine direction (MD) and the transverse direction (TD), to align the polymer chains [12,28,29], which was able to adjust the mechanical behavior of the films as a result. For example, the mechanical properties of polyamide 6/ethylene-vinyl alcohol (PA6/EVOH) copolymer films were improved by bidirectional stretching, and the obtained films exhibited excellent mechanical properties when the stretching ratio was 3 × 3 [30]. Furthermore, it has been reported that via biaxial stretching, the transparency of the films can also be improved via adjusting the orientation of the polymer chains. For instance, the DOW chemical company reported transparent tenter-frame biaxially oriented polyethylene (TF-BOPE) films, which displayed high optical performance with the light transmittance of about 90% at a thickness of 25–40 μm [31]. In addition, the optical performance of the BOPLA was also improved, and its transmittance was 87.0% [27].

Compared with other copolymers with good optical properties (such as 1,3-cyclohexane bis(methylamine) (1,3 BAC), para-bis(4-amino-3-methylcyclohexyl)methane (MACM), parabis(4-aminocyclohexyl)propane (PACP), etc.), PACM has not only excellent wear resistance, chemical resistance, and stress cracking resistance but also good thermal stability and processing formability [32].

Therefore, in this study, to improve the transparency of the PA66 without trade-off of its mechanical performance, we proposed using the melting copolymerization method to prepare the PA66/PACM6 copolymer and investigated the effect of the composition on the transparency, crystallization behavior, and mechanical performance of the copolymer. Subsequently, the obtained copolymers were cast into films on a cooling roller and subjected to biaxial stretching. By differential scanning calorimetry (DSC), wide-angle X-ray diffraction (WAXD), and UV–visible (UV-vis) spectrophotometry, examining the crystalline phase transitions and optical properties, it was clearly clarified that the relationship between the crystallinity and overall properties of PA66/PACM6 copolymers changes based on adjusting their compositions and tensile ratios. This practical investigation offers theoretical perspectives that can enhance large-scale manufacturing methods and broaden the range of applications for PA66/PACM6 copolymers.

## 2. Experimental

### 2.1. Materials

Raw material polyamide 66 salt was purchased from BASF (China) Co., Ltd., Shanghai, China with a purity of 99.9%. The adipic acid (AA, purity of 99.8) was bought from Zhejiang Huafeng New material Co., Ltd., Ruian China. 4,4′-Diaminodicyclohexylmethane (PACM) was purchased from Huntsman Corporation, Salt Lake City, UT, USA. Ethanol (purity of 95%) was bought from Hunan Xiangyikang Pharmaceutical Co., Ltd., Yiyang, China.

### 2.2. Preparation of Copolymers

First, a certain amount of AA was added into ethanol at 80 °C with a mass ratio of 20 wt%. Then, the PACM liquid was slowly added into the AA solution drop by drop until the opaque solution became clear, and the pH was controlled at 7.2–7.5. Following that, the clear solution was cooled down to room temperature, and the PACM6 solid was successfully precipitated from the solution, which was then washed with ethanol three times and dried at 80 °C in a vacuum oven for 6 h. The yield was about 85%.

**Figure 1:**
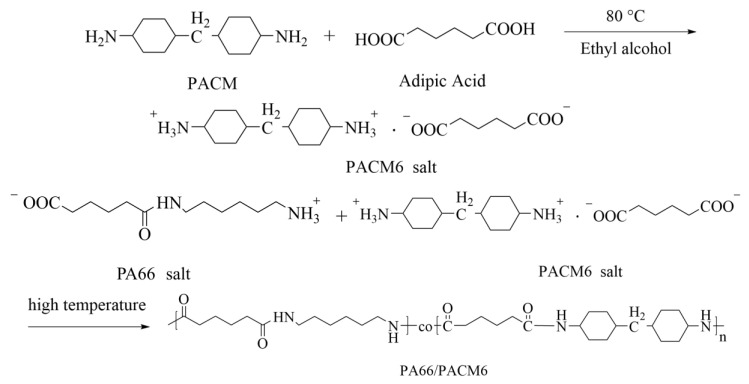
The synthesis process of PA66/PACM6 copolymer.

Second, the obtained PACM6 salt and the PA66 salt were introduced into a polymerization reactor (Figure 1). The weight of PACM6 was varied from 0% to 40 wt% of the solids. After that, a certain amount of the deionized water at 2 wt% of the solids was added into the reactor, which was evacuated to 0.1 MPa. To eliminate air, the reactor was purged with high-purity nitrogen gas three times. The reactor was subsequently heated to 210 °C, while the pressure was maintained at about 1.5–2.0 MPa for 2 h. Then, the temperature was raised to 270 °C, and a controlled venting process was employed to remove the water vapor and other gases from the reactor. Next, a gradual vacuum was applied to decrease the system pressure to −0.01 MPa. At the end of the reaction, nitrogen gas was introduced, and the product was discharged through the opened bottom valve. The resulting material was granulated to obtain the PA66/PACM6 resin, which was then dried in a vacuum oven and stored. The obtained copolymer was named as PA66/PACM6 (*n* wt%) based on the weight ratio of the PACM6 of the PA66 matrix, where *n* represents the weight ratio of PACM6 in the copolymer. For example, PA66/PACM6 (20 wt%) meant that the weight ratio of PACM6 was 20 wt% of the PA66 in the copolymer during the synthesis.

### 2.3. Preparation of Casting Films and Biaxially Oriented Films

**Figure 2:**
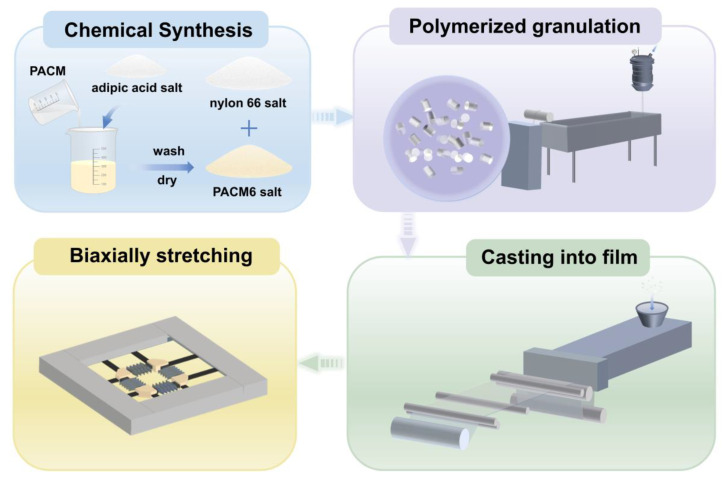
The complete preparation process of BO-PA66/PACM6 films.

The PA66/PACM6 copolymer films with a thickness of 200 μm were prepared using a casting machine (Guangzhou Putong Experimental Analytical Instrument Co., Ltd. (Guangzhou, China), FDHU-35). Based on the mechanical performance of the casting films of the copolymers, PA66/PACM6 (20 wt%) was chosen to perform the biaxially oriented process. First, the obtained PA66/PACM6(20 wt%) casting film was cut into square samples with an area of 100 × 100 mm^2^ and placed on a stretching apparatus of the biaxial machine (Bruckner Machinery Co., Ltd. (Suzhou, China), KARO 5.0). The longitudinal (MD) and transverse (TD) directions meant that the MD and the TD were parallel and perpendicular to the direction of the casting process, respectively. Before stretching, the perimeter of the samples was secured using five clamps. Subsequently, the film was heated to 165 °C for 1 min and underwent simultaneous biaxial stretching at a constant rate of 100 mm/s to different stretching ratios of 1 × 1 to 3 × 3. For 1 × 1, it meant that the casted film was not stretched. For 3 × 3, the casted film was stretched to three times that of the original length in the MD and TD direction. After stretching, the biaxially oriented film was kept at 160 °C for 1 min to be fixed. It is notable that the unstretched film also underwent the same heating treatment for comparison. The biaxially oriented films were named as BO-PA66/PACM6 later. The complete preparation process is shown in Figure 2.

### 2.4. Measurements

Nuclear magnetic resonance (NMR) spectra were recorded using a Bruker AVANCE AV 400 spectrometer (Germany Bruker Technology Co., Ltd. Saarbrucken, Germany). The samples were thoroughly dried, and deuterated trifluoroacetic acid was selected as the solvent for ^1^H NMR characterization.

Fourier transform infrared (FT-IR) spectra were obtained using a BRUKER TENSOR 27 FT-IR spectrometer(Germany Bruker Technology Co., Ltd. Saarbrucken, Germany) Thin films were measured in an attenuated total reflection mode with a wavelength range of 400–4000 cm^−1^.

The crystallinity of the copolymer granules and the biaxially stretched film was investigated via experiments under a nitrogen atmosphere using a Q20 differential scanning calorimetry (DSC) instrument (TA, New Castle, DE, USA). The sample weight was about 4 mg. Initially, the samples were heated from 25 °C to 300 °C and held at 300 °C for 3 min to eliminate any residual thermal history. Subsequently, they were cooled down to 25 °C at a rate of 10 °C/min and then reheated to 300 °C at the same rate. The crystallinity (*X_c_*) of the samples was calculated using the equation [26]

(1) $$X_{c}=\frac{\Delta H_{m}}{Ø\times \Delta H_{m}^{0}}\times 100\%$$

where Δ*H_m_* is the melting enthalpy, Ø is the mass fraction of PA66, and $\Delta H_{m}^{0}$ is the melting enthalpy of 100% crystalline PA66 (206 J/g) [33].

Thermogravimetric analysis (TGA) of the copolymers was examined on a Q50 TGA instrument (TA, New Castle, DE, USA), which was heated from 25 °C to 600 °C at a heating rate of 10 °C/min in a nitrogen atmosphere.

Wide-angle X-ray diffraction (WAXD) measurements were performed using Cu Kα radiation (λ = 0.154 nm) on the Xeuss 2.0 system by Xenocs SA, Grenoble, France. Two-dimensional patterns were recorded on a single-photon counting detector (Pilatus 300K, DECTRIS, Baden, Switzerland), with a sample-to-detector distance set at approximately 176 mm. The exposure time for each sample was 30 s.

The optical performance of the films was evaluated using a UV-2600 UV–Visible spectrophotometer (Shimadzu Corporation, Kyoto, Japan) and a WGT-S Haze measuring instrument (Jinan Languang electromechanical Technology Co., Ltd., Jinan, China) with a size of 10 mm × 10 mm × 2 mm ranging from 350 nm to 800 nm.

The mechanical testing was carried out on a universal materials testing machine (ETM 502 B-EX, Wance Testing Machine Shenzhen Ltd., Shenzhen, China) at room temperature (23 °C, 50% relative humidity). The testing speed was set as 50 mm/min. Five samples of each group were tested to obtain the tensile strength and elongation at break. The sample was cut into a dumbbell shape 75 mm in length and 4 mm in width.

## 3. Results and Discussion

### 3.1. Structural Characterization Analysis

**Figure 3:**
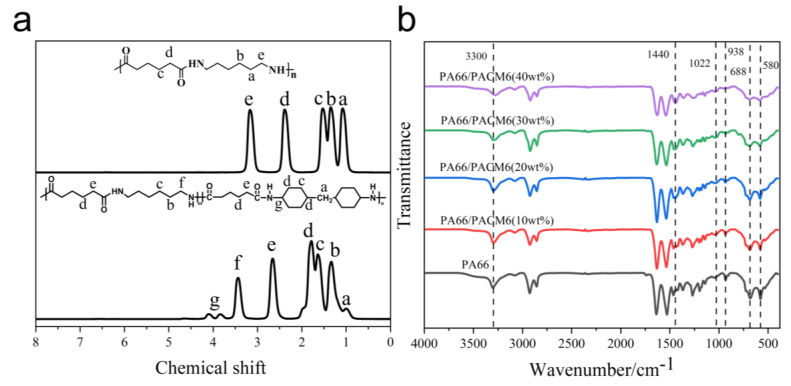
(**a**) ^1^H−NMR spectra of PA66 and PA66/PACM6. (**b**) FT−IR spectra of PA66/PACM6 with different PACM6 content.

Figure 3a displays the proton nuclear magnetic resonance (^1^H NMR) spectrum of PA66 and PA66/PACM6. The peak at 1.01 corresponded to the protons on the methylene group attached to the cyclohexyl ring, and the peak at 3.84 and 4.09 was ascribed to the protons on the cyclohexyl group and the amino-linked submethyl group. The chemical shift peaks on the spectrum correspond one-to-one with the polymer structure, confirming that the synthesized compound is the target product, PA66/PACM6 [15].

In Figure 3b, it can be found that the peak located around 3300 cm^−1^ ascribed to the stretching vibration of N–H was shifting toward lower wavenumbers in the spectra of PA66/PACM6 compared with that of PA66. Moreover, the intensity of the peak was also decreased with the increase in PACM6 in the copolymer. These results indicated that after loading PACM6, the hydrogen bonds between the PA66 molecules were decreased due to the cycloaliphatic ring structure in the polymer chains [34,35], which might further affect the crystallization of the PA66 matrix. Moreover, the absorption peak at 1470 cm^−1^ corresponding to the stretching vibrations of the methylene groups adjacent to the amide group in PA66 was shifted downward to 1440 cm^−1^ for PA66/PACM6 (40 wt%) [36,37]. These suggested that the molecular chains of PA66 have endured an obvious transition after being loaded into PACM6 from the monoclinic α-crystalline form to the pseudo-hexagonal γ-crystalline form [38].

Furthermore, the sharp peaks at 933 cm^−1^, 688 cm^−1^, and 580 cm^−1^ originated from the bending vibration of C–CO, and the out-of-plane bending vibrations of NH and CO corresponded to the regular α-crystalline form in PA66. However, the broad peak at 1022 cm⁻^1^ was related to the amorphous structure of polyamide [37]. Compared with the original PA66, it was clear that the intensity of sharp peaks from the regular crystal form, which were gradually decreased with the peak intensity of the amorphous structure, was enhanced as PACM6 increased in the copolymer, indicating that the introduction of PACM6 weakened the crystalline peak, which significantly reduced the regularity of the crystalline structure [34].

### 3.2. Crystallization Behavior Analysis

**Figure 4:**
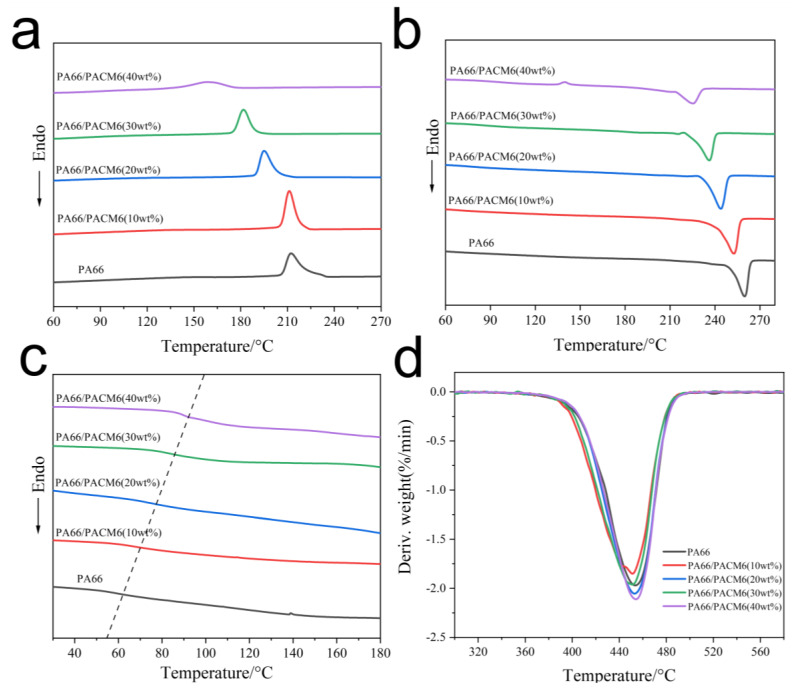
The DSC curves of PA66 and PA66/PACM6 copolymers: (**a**) cooling segment, (**b**) second heating segment (10 °C/min), and (**c**) second heating segment (20 °C/min). (**d**) The DTG curves of PA66/PACM6 copolymer.

The thermal results of the copolymers are displayed in Figure 3 and Table S1. As displayed in Figure 4a, the crystallization peak of the copolymer exhibited a significant reduction in the crystallization temperature compared with the PA66 matrix due to the presence of the rigid cyclic structure in PACM6 that hindered the motions of the polymer chains during the crystallization process, which further affected the crystallization ability of molecular chains as a result [39]. Moreover, the crystallinity of the synthesized copolymer decreased from 28.3% to 15.6% (Figure 4b and Table S1), indicating that the crystallizing ability of the copolymer was much lower than that of PA66, which was ascribed to the random polymerization of PA66/PACM6 that reduced the regularity of the molecular chain alignment. On the other hand, the glass transition temperature of the copolymer was also increased with the increase in PACM6 from 63.3 °C for PA66 to 92.7 °C for PA66/PACM6 (40 wt%) as observed in Figure 4c. That was caused by the introduction of PACM-6, which contained large molecular size and complex structure and led to the increase in the rigidity of the molecular chains, which affected the motion of the polymer chains at low temperature. Furthermore, we also performed TGA to observe the thermal stability of the obtained copolymers, and the results are displayed in Figure 4d and Figures S1 and S2. The thermal decomposition curve and the derivative thermogravimetric (DTG) curve of the copolymer displayed a single weight loss peak in the temperature range of 350 to 600 °C, indicating that the thermal decomposition of PA66/PACM6 in nitrogen exhibited a single thermal degradation. Moreover, compared with PA66, the change in the onset decomposed temperature of the copolymers was negligible, indicating that the introduction of PACM6 did not compromise the thermal stability of PA66.

**Figure 5:**
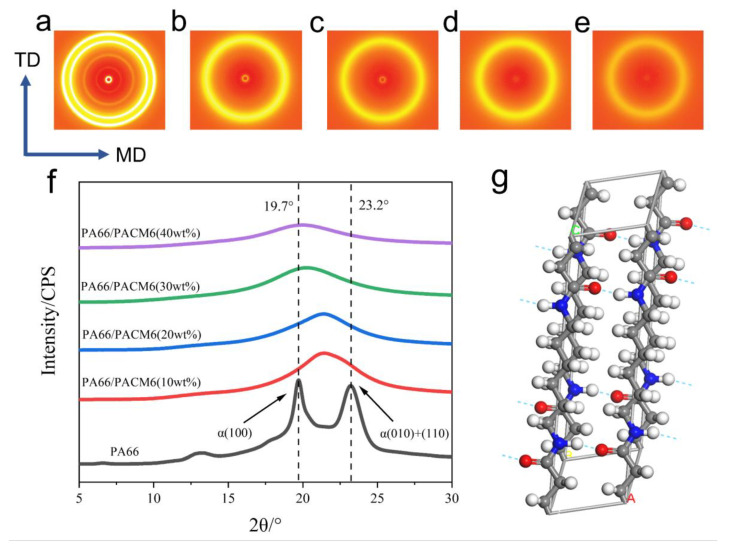
WAXD patterns of different ratios of PA66/PACM6 copolymers film: (**a**) 0%, (**b**) 10%, (**c**) 20%, (**d**) 30%, and (**e**) 40%. (**f**) 1D WAXD patterns of PA66/PACM6 copolymers films. (**g**) 3D diagram of PA66 α crystal form (black for carbon atoms, red for oxygen atoms, and white for hydrogen atoms).

Figure 5a–e display the two-dimensional WAXD images of the copolymer films. It is evident that PA66 exhibited two distinct strong diffraction rings. However, as PACM6 was introduced, these distinct rings gradually transformed into a more diffused and blurry one, indicating that there was a clear transition in the crystallization structure of the copolymer contributed by the introduced PACM6. To further investigate the crystalline transformation in the copolymer, 1D WAXD curves were obtained by integrating the 2D WAXD pattern, and the result is shown in Figure 5f. It can be found that PA66 displayed two characteristic diffraction peaks at 19.7° and 23.2°, representing (100), (010), and (110) crystallographic planes that were ascribed to the α-crystalline form of PA66. With the presence of PACM6 in the copolymer, the double peaks of PA66 transformed into a wider single peak with the peak position shifting to lower degrees as PACM6 increased, which indicated that the α-crystalline form in PA66 has transformed into γ-crystalline form with a less regular lattice structure caused by the disordered arrangement of the polymer chains [40], which was also consistent with the FTIR and DSC results.

This phenomenon can be explained by the fact that during the formation of PA66, the PA66 molecular chains in the primary crystalline structure of the α-type form adopted a fully extended planar zigzag conformation parallel to the c-axis direction. The lattice parameters for the monoclinic *α*-type crystalline structure are a = 0.49 nm, b = 0.54 nm, c = 1.72 nm, *α* = 48°, *β* = 77°, and *γ* = 63° (Figure 5g). On the other hand, the *γ*-type crystal form in a pseudo-hexagonal shape is the other stable crystalline structure of PA66, which was formed with shorter molecular chains. The introduction of PACM6 disrupted the crystalline regularity of PA66, leading to a reduction in the copolymer’s crystallinity and resulting in a transition from the monoclinic *α*-type to the pseudo-hexagonal *γ*-type crystalline structure [41,42].

### 3.3. Optical Performance Analysis

**Figure 6:**
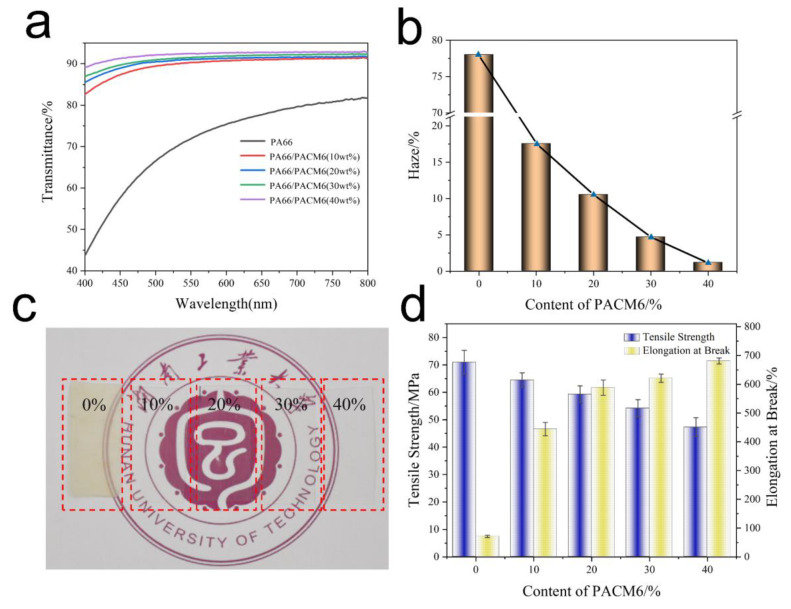
Optical property of PA66/PACM6 copolymer film materials. (**a**) Transmittance, (**b**) haze, and (**c**) the photos of PA66/PACM6 to exhibit its transparency (The non-English term is the Hunan University of Technology). (**d**) The strength and elongation at the break of PA66/PACM6 copolymer films.

The transmittance of the synthesized PA66/PACM6 resin was assessed, and the results are shown in Figure 6a and Table S2. It was found that the transmittance of the material was significantly improved with the increase in PACM6 in the copolymer, which achieved 92.5% within the visible light range when the PACM6 content was 40 wt% (at 550 nm). This high transmittance was contributed by the structural arrangement of polymer chains. In detail, after introducing PACM6, the crystallinity of PA66 and the hydrogen bonding interaction of the molecular chains (exhibited in the FTIR results) were greatly reduced, which was helpful for the improvement in the transparency of the resultant copolymers. Figure 6b illustrates the haze of PA66/PACM6 copolymers. It was observed that as the PACM6 content increased, the haze of the copolymer significantly decreased. When the PACM6 content was 40 wt%, the haze of the PA66/PACM6 films was 1.2%, indicating greater clarity and transparency of the copolymer. In contrast, for the pure PA66, the high crystallinity as well as the regular crystal blocked the pass of the visible light, which exhibited a much lower transparency (Figure 6c).

### 3.4. Mechanical Properties Analysis

The mechanical performance of the copolymers was evaluated, and the results are shown in Figure 6d and Table S3. It was found that the addition of PACM6 in the copolymer greatly adjusted the mechanical performance of the obtained films. The mechanical strength was gradually decreased with a sharp increase in the elongation at break compared with PA66, which was contributed by the enhanced mobility of the molecular chains upon stretching due to the weakened hydrogen bonding interactions between the PA66 segments in the copolymers via the introduced PACM6 upon stretching. Moreover, it was noticed that when PACM6 was 20 wt%, the resultant copolymer exhibited a relatively high mechanical strength with the elongation at a break of 588.3%, which was chosen to perform the biaxially oriented experiment.

### 3.5. Crystallization Behavior of BO-PA66/PACM6 Films

**Figure 7:**
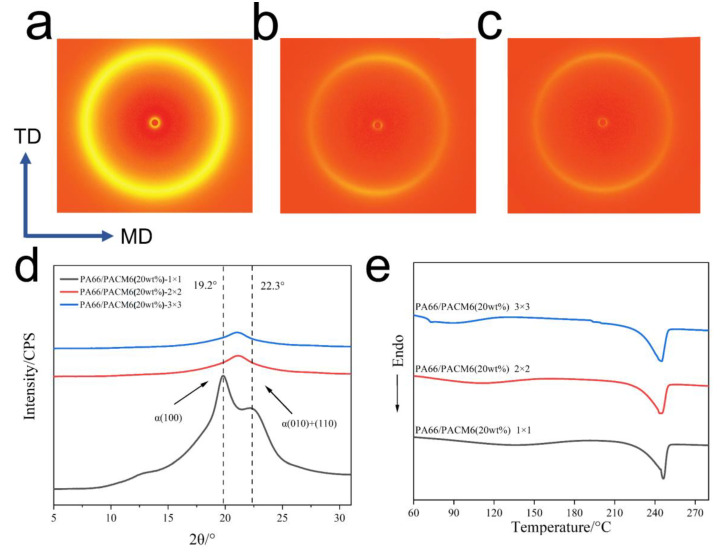
The WAXD patterns of the BO-PA66/PACM6 films upon different stretching ratios in the MD–TD plane: (**a**) 1 × 1, (**b**) 2 × 2, and (**c**) 3 × 3. (**d**) 1D WAXD patterns of the BO-PA66/PACM6 films at different stretching ratios in the MD–TD plane. (**e**) DSC curves of the BO-PA66/PACM6 films with different stretching ratios from first heating.

The 2D WAXD patterns of the biaxially stretched PA66/PACM6 films are shown in Figure 7a–c. In the case of the heating-treated film (Figure 7a), the intensity of the diffraction ring was noticeably enhanced compared with the untreated one (Figure 5c), indicating an increase in the crystallinity of the copolymer film. With an increase in the stretching ratio, the diffraction signals in the direction of MD became stronger than those in the TD direction accompanied by the transform into non-uniform rings with the (010) and (110) lattice planes evolving from two semicircles into two shorter arcs, suggesting an increase in the crystalline orientation of the BO-PA66/PACM6 film [30,43]. When the stretching ratio was further increased to 3 × 3, the intensity of the diffraction ring was further decreased. Further analysis was provided in the 1D WAXD curves (Figure 7d). After the heating treatment, the copolymer film (1 × 1) exhibited two characteristic peaks (located at 19.2° and 22.3°) ascribed to the *α*-crystalline form. Compared with the untreated film (Figure 5f), the reappearance of the *α-* crystalline form was caused by the rearrangement of the polymer chains during the heating treatment. However, after being biaxially stretched, the repeated α- crystalline form was transformed into γ-form. The phase transition induced by being biaxially stretched is a process of crystallization destruction and recrystallization. The crystal size of the recrystallized γ-form may be smaller than the original crystal size of the α-form, so the peak intensity at α-form (100) and (010) + (110) is sharper than the peak intensity of the γ-form that appears on the stretched film [44,45,46]. Moreover, the optical properties of the film are improved with the reduction in the crystal size [47]. The DSC results of the BO-PA66/PACM6 films at different stretching ratios are shown in Figure 7e. The broad peak in the range of 75–150 °C was attributed to the evaporation of the absorbed moisture during the casting process. It can be found that the melting temperature and the crystallinity of BO-PA66/PACM6 were slightly increased with the increase in the stretching ratio (Table S4) due to the more regular arrangement of the polymer chains upon stretching.

### 3.6. The Optical Performance Analysis of BO-PA66/PACM6 Films

**Figure 8:**
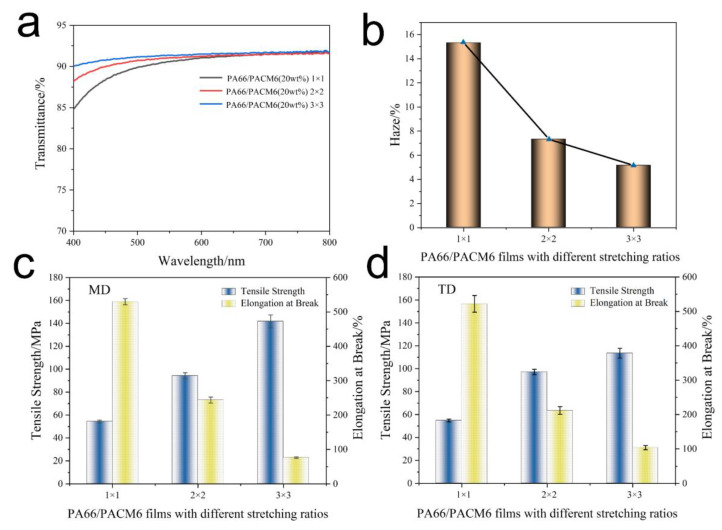
The optical performance of the BO-PA66/PACM6 copolymer films with different stretching ratios: (**a**) transmittance and (**b**) haze. Tensile strength and elongation at break: (**c**) MD and (**d**) TD of the BO-PA66/PACM6 copolymer films.

The optical performance of BO-PA66/PACM6 was investigated, and the results are exhibited in Figure 8a,b and Figure S3 and Table S5. It can be found that the transmittance of BO-PA66/PACM6 was gradually enhanced as the biaxial stretching ratio increased. It can be found that after performing biaxial stretching, although the crystallinity of BO-PA66/PACM6 was slightly increased, it still showed good transparency. This might be because the grain size formed by stress-induced crystallization is smaller than the visible wavelength, and thus the crystallized samples showed good transparency and the transmittance slightly increased with an increasing stretching ratio [27]. When the stretching ratio was 3 × 3, the film became nearly fully transparent with a transmittance of 91.6% (at 550 nm), which was much higher than those in the previous reports (Table S6). Figure 8b illustrates the relationship of the haze in relation to the biaxial stretching ratio. It was observed that after biaxially stretching, there was a notable decrease in haze as low as 5.2% with the stretching ratio of 3 × 3. The possible reasons might be as follows: the arrangement of the polymer molecular chains became more ordered upon biaxial stretching, which would reduce the random scattering between molecules and enable the light to pass through the film more directly and enhance the transparency as a result. Additionally, the biaxially stretching changes the surface roughness of the film mainly due to the more or less preferential orientation of molecular chains under the action of tensile stress. With the increase in the tensile ratio, the orientation of the sample may be relatively uniform, forming a more evenly distributed structure [27,48,49], which further decreased the light scattering and enhanced the transparency of the films.

### 3.7. Mechanical Properties of the BO-PA66/PACM6 Films

The mechanical performance of BO-PA66/PACM6 films at different stretching ratios was investigated, and the results are displayed in Figure 8c,d and Table S7. It can be found that the mechanical strength greatly enhanced, which was 141.8 MPa and 113.6 MPa in the MD and TD direction, respectively, when the stretching ratio was 3 × 3. However, its corresponding elongation at break exhibited an opposite trend compared with that of mechanical strength. This obvious enhancement in the mechanical strength of the films was attributed to the directional alignment of polymer chains during biaxial stretching, which would promote a more parallel arrangement of polymer chains, leading to an increase in crystallinity and thus improving the tensile strength. Moreover, the stretching was favorable for reducing the defects, voids, and irregularities, which resulted in a more uniform film and further enhanced the tensile strength [27,47,48,49]. However, the highly aligned polymer chains were too rigid to transfer the internal stress upon stretching, leading to the decreased ductility of the films as a result.

## 4. Conclusions

In this study, the optical performance of PA66 was successfully improved via copolymerizing it with PACM6. It was found that after introducing PACM6 into PA66, the crystal form as well as the crystallinity of PA66 was effectively adjusted, which revealed a clear transition from the α to γ crystalline phase with an increase in the PACM6 content. The transmittance and haze of the copolymer were highly improved, with a transmittance and haze of as high as 92.8% and as low as 1.2%, respectively. Compared with the transparent polyamide films reported so far (Table S8), the optical properties of PA66/PACM6 films are more excellent. Moreover, the ductility of PA66 was significantly enhanced via the loading of PACM6 in the copolymer, which was improved by 859% compared with that of pure PA66. Furthermore, to further enhance the optical performance of the PA66/PACM6 film at a lower content of PACM6 without a trade-off in the mechanical strength, biaxial stretching was further employed to prepare the BO-PA66/PACM6 films, which was highly favorable for the improvement in the optical performance as well as the mechanical strength of the prepared films, achieving up to 141.8 MPa. Compared with PVA, PMMA, and other common trans-parent semi-crystalline polymers (Table S9), the resultant films in this study displayed higher tensile strength, which makes them have a huge application prospect in the packaging. Therefore, the approach proposed in this study provided a new perspective for the preparation of highly transparent polyamide films.

## Data Availability

Data are contained within the article and Supplementary Materials.

## Supplementary Materials

The following supporting information can be downloaded at: https://www.mdpi.com/article/10.3390/polym16050599/s1. Figure S1. The TGA curves of PA66/PACM6 copolymer. Figure S2. The detail enlargement of PA66/PACM6 TGA curve. Table S1. Thermal performance parameter on PA66 and PA66/PACM6 films. Table S2. Optical property parameters on PA66 and PA66/PACM6 films. Table S3. Mechanical property parameters on PA66 and PA66/PACM6 films. Table S4. DSC parameters of PA66/PACM6 (20 wt%) films with different stretching ratios from first heating thermogram curves. Figure S3. The picture of biaxially tensile PA66/PACM6 films with different stretching ratios. Table S5. Optical property parameters of PA66/PACM6 (20 wt%) films with different stretching ratios. Table S6. Mechanical property parameters of PA66/PACM6 (20 wt%) films with different stretching ratios (MD). Table S7. Mechanical property parameters of PA66/PACM6 (20 wt%) films with different stretching ratios (TD). Table S8. Optical property comparison with recently reported studies. Table S9. Comparison of tensile strength between PA66 and common transparent semi-crystalline polymeric materials. References [7,9,17,47,50,51,52,53,54,55,56] are cited in the supplementary materials.
