# Supplementary material for: An Intrinsically Transparent Polyamide Film with Superior Toughness and Great Optical Performance
Source: Polymers (Basel). 2024 Feb 22;16(5):599. doi: 10.3390/polym16050599 (PMC10935283; doi:10.3390/polym16050599)
Supplement: Supplementary file 1 [file polymers-16-00599-s001.zip › polymers-2856974-supplementary.pdf]

Supporting Information

An Intrinsically Transparent Polyamide Film with Superior Toughness and Great Optical Performance

Jianlin Li[1], Yong Yi[1], Liang Chen[2], Chunhua Wang[1], Weijian Lu[1], Mingxi Liao[1], Xin Jing[1], Wenzhi Wang[1*]*

[1] National & Local Joint Engineering Research Center for Advanced Packaging Material and Technology, Hunan University of Technology, Zhuzhou, Hunan, 412007, China

[2]Haiyang Technology Co., LTD., Taizhou 225300, China

***Corresponding Authors:**

Wenzhi Wang, E-mail: wangwenzhi@hut.edu.cn

Xin Jing, E-mail: jingxin@hut.edu.cn

This supporting information contains:

– 14pages (S1–S14)

– 9 tables (Table S1–S9)

– 3 figures (Figure S1–S3)

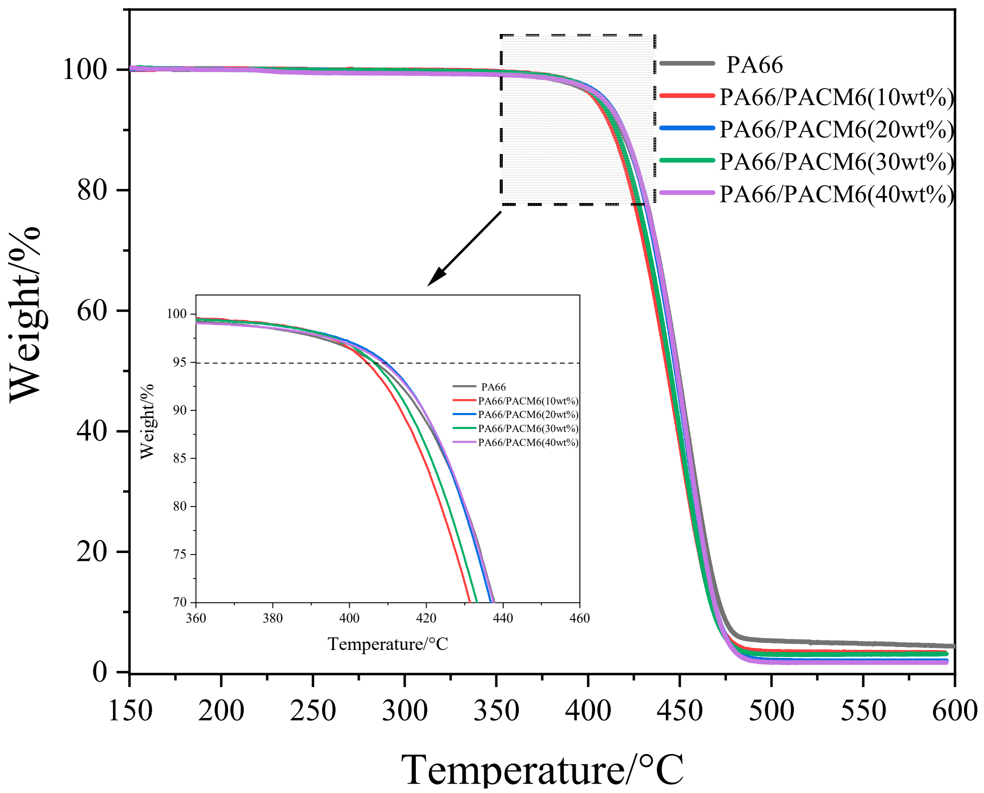

Figure S1. The TGA curves of PA66/ PACM6 copolymer

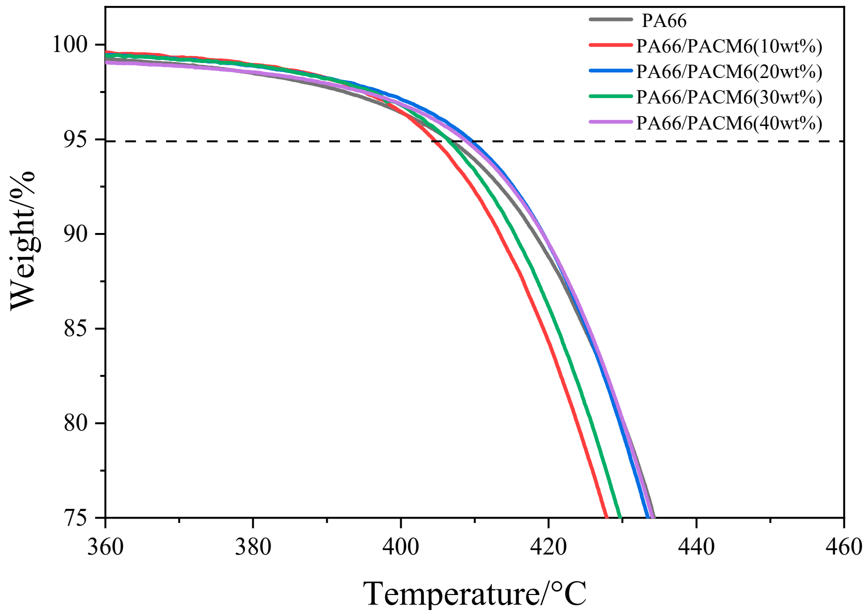

Figure S2. The detail enlargement of PA66/PACM6 TGA curve.

Table S1. Thermal performance parameter on PA66 and PA66/PACM6 films

Sample	T_c(°C)	T_m(°C)	ΔH_m(J/g)	X_c(%)	T_g(°C)	$T_{5\%}$(°C)
PA66	212.0	259.8	58.2	28.3	63.3	406.5
PA66/PACM6(10wt%)	211.0	253.9	52.0	28.0	72.1	404.5
PA66/PACM6(20wt%)	196.2	247.0	44.7	27.1	79.3	409.4
PA66/PACM6(30wt%)	193.8	236.2	33.7	23.4	86.2	406.3
PA66/PACM6(40wt%)	159.1	225.2	19.3	15.6	92.7	408.6

Table S2. Optical property parameters on PA66 and PA66/PACM6 films

Sample	Transmittance /% (at 550 nm)	Haze/%
PA66	81.8	78.1
PA66/PACM6(10wt%)	90.3	17.6
PA66/PACM6(20wt%)	91.1	10.5
PA66/PACM6(30wt%)	91.5	4.7
PA66/PACM6(40wt%)	92.5	1.2

Table S3. Mechanical property parameters on PA66 and PA66/PACM6 films

Sample	Tensile Strength/MPa	Elongation at Break/%
PA66	71.0 ± 4.4	71.0 ± 3.8
PA66/PACM6(10wt%)	64.5 ± 2.7	444.2 ± 23.0
PA66/PACM6(20wt%)	59.3 ± 3.1	588.3 ± 26.5
PA66/PACM6(30wt%)	54.2 ± 3.1	621.0 ± 14.3
PA66/PACM6(40wt%)	47.3 ± 3.4	680.8 ± 10.6

Table S4. DSC parameters of PA66/PACM6(20wt%) films with different stretching ratios from first heating thermogram curves.

Sample	ΔH_m(J/g)	X_c(%)
PA66/PACM6(20wt%)-1×1	49.6	30.1
PA66/PACM6(20wt%)-2×2	52.9	32.1
PA66/PACM6(20wt%)-3×3	54.7	33.2

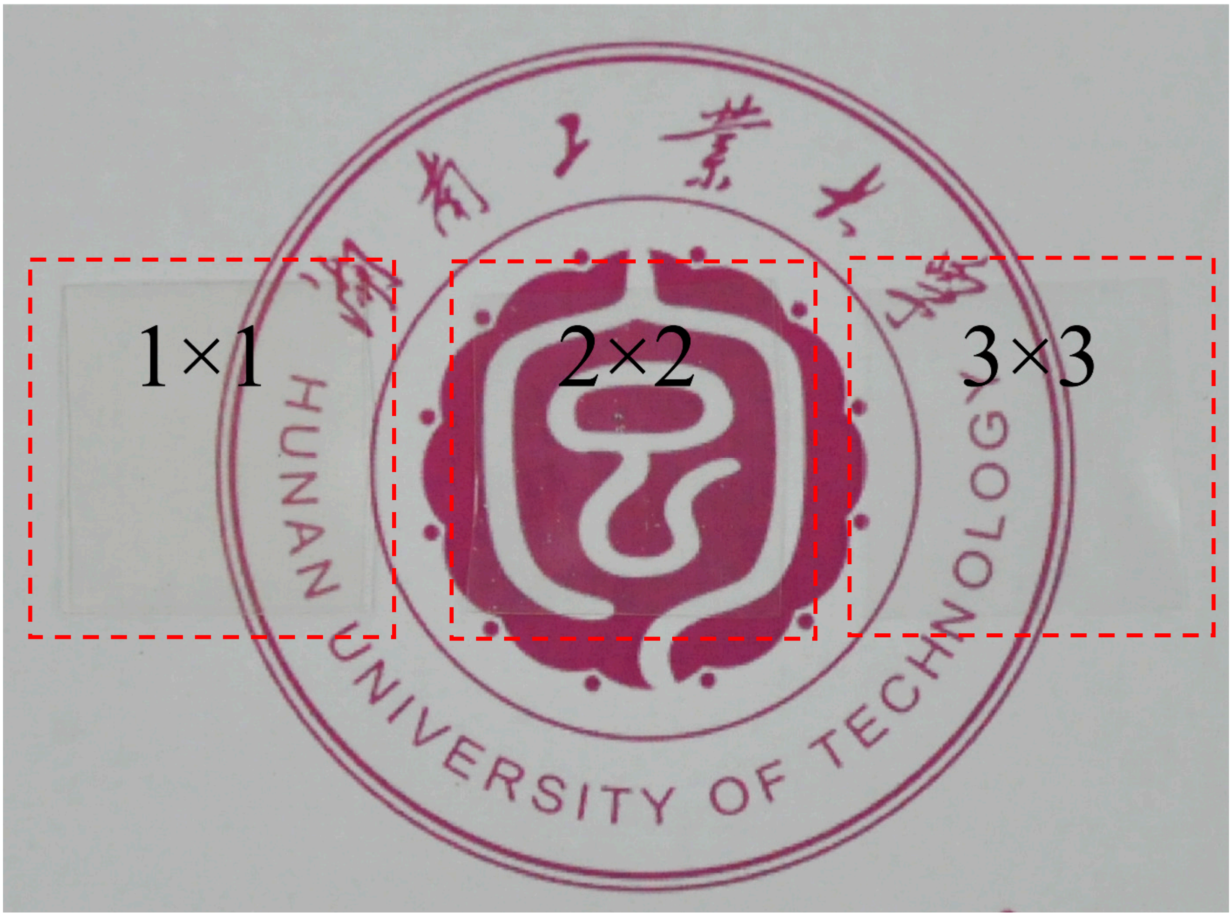

Figure S3. The picture of biaxially tensile PA66/PACM6 films with different stretching ratios. (The non-English term is the Hunan University of Technology)

Table S5. Optical property parameters of PA66/PACM6(20wt%) films with different stretching ratios

Sample	Transmittance/% (at 550 nm)	Haze/%
PA66/PACM6(20wt%)-1×1	90.6	15.3
PA66/PACM6(20wt%)-2×2	91.3	7.3
PA66/PACM6(20wt%)-3×3	91.6	5.2

Table S6. Mechanical property parameters of PA66/PACM6(20wt%) films with different stretching ratios (MD)

Sample	Tensile Strength/MPa	Elongation at Break/%
PA66/PACM6(20wt%)-1×1	54.4 ± 1.1	529.7 ± 9.0
PA66/PACM6(20wt%)-2×2	94.3 ± 2.6	243.7 ± 8.7
PA66/PACM6(20wt%)-3×3	141.8 ± 5.6	76.2 ± 2.1

Table S7. Mechanical property parameters of PA66/PACM6(20wt%) films with different stretching ratios (TD)

Sample	Tensile Strength/MPa	Elongation at Break/%
PA66/PACM6(20wt%)-1×1	54.8 ± 1.1	522.1 ± 24.2
PA66/PACM6(20wt%)-2×2	97.1 ± 2.4	211.9 ± 11.0
PA66/PACM6(20wt%)-3×3	113.6 ± 5.6	103.6 ± 6.0

Table S8. Optical property comparison with recently reported studies.

Materials	Transmittance/%	Ref.
**PA66/PACM6 films**	**91.4-92.8%**	**This work**
Polyamide-imides films with different monomer sequences	59.3-89.7%	Polymer, 2020[1]
PA-6/PMMA nanocomposite films	86%	Polymer, 2021[2]
Transparent Polyamide 11 Nanofibers Films	38-77%	Advanced Functional Materials, 2008[3]
The PMMA/nylon-6 composite films	80-85%	Composites Science and Technology, 2013[4]
Nylon-6 nanofiber polyurethane composites	85-89%	ACS Applied Materials & Interfaces,2012[5]
Transparent polyamide-imide films	75.3-87.7%	Polymer, 2020[6]
Organ-soluble polyamide films with bulky alicyclic pendent group	88-91%	Polymer, 2007[7]
Transparent random and multi-block polyamide-imide films	84-87%	European Polymer Journal, 2021[8]

Table S9. Comparison of tensile strength between PA66 and common transparent semi-crystalline polymeric materials.

Sample	Tensile Strength/MPa
Nylon 66 (PA66)	50-80[9]
2, 2, 6, 6-tetramethylpiperidine-1-oxyl (TEMPO)-oxidized bacterial cellulose powder) (TOBCP)	30-80[10]
Poly (methyl methacrylate) (PMMA)	50-80[11]
Polyvinyl alcohol (PVA)	20-40[11]
Polycarbonate (PC)	60-80[11]

References

[1] MUSHTAQ N, WANG Q, CHEN G, et al. Synthesis of polyamide-imides with different monomer sequence and effect on transparency and thermal properties [J]. Polymer, 2020, 190: 122218.
[2] LI B Y, WEI S, XUAN H Y, et al. Tailoring fineness and content of nylon-6 nanofibers for reinforcing optically transparent poly(methyl methacrylate) composites [J]. POLYMER COMPOSITES, 2021, 42(7): 3243-52. https://doi.org/10.1002/pc.26054
[3] HAVEL M, BEHLER K, KORNEVA G, et al. Transparent Thin Films of Multiwalled Carbon Nanotubes Self-Assembled on Polyamide 11 Nanofibers [J]. Advanced Functional Materials, 2008, 18(16): 2322-7.
[4] JIANG S, GREINER A, AGARWAL S. Short nylon-6 nanofiber reinforced transparent and high modulus thermoplastic polymeric composites [J]. Composites Science and Technology, 2013, 87: 164-9.
[5] JIANG S, DUAN G, HOU H, et al. Novel layer-by-layer procedure for making nylon-6 nanofiber reinforced high strength, tough, and transparent thermoplastic polyurethane composites [J]. ACS Applied Materials & Interfaces, 2012, 4(8): 4366-72.
[6] LAO H, MUSHTAQ N, CHEN G, et al. Transparent polyamide-imide films with high Tg and low coefficient of thermal expansion: Design and synthesis [J]. Polymer, 2020, 206: 122889.
[7] LIAW D-J, CHEN W-H, HU C-K, et al. High optical transparency, low dielectric constant and light color of novel organosoluble polyamides with bulky alicyclic pendent group [J]. Polymer, 2007, 48(22): 6571-80.
[8] LAO H, MUSHTAQ N, CHEN G, et al. Synthesis and properties of transparent random and multi-block polyamide-imide films with high modulus and low CTE [J]. European Polymer Journal, 2021, 153: 110512.
[9] YANG B, LENG J, HE B, et al. Influence of fiber length and compatibilizer on mechanical properties of long glass fiber reinforced polyamide 6,6 [J]. Journal of Reinforced Plastics and Composites, 2012, 31(16): 1103-12. https://doi.org/10.1177/0731684412454199
[10] RAHMADIAWAN D, SHI S-C, ABRAL H, et al. Comparative Analysis of the Influence of Different Preparation Methods on the Properties of TEMPO-Oxidized Bacterial Cellulose Powder Films [J]. Journal of Natural Fibers, 2024, 21(1): 2301386. https://doi.org/10.1080/15440478.2023.2301386
[11] LIN Y, BILOTTI E, BASTIAANSEN C W M, et al. Transparent semi-crystalline polymeric materials and their nanocomposites: A review [J]. Polymer Engineering & Science, 2020, 60(10): 2351-76. https://doi.org/https://doi.org/10.1002/pen.25489
